# Notes on Preparations for the Sick

**Published:** 1895-03

**Authors:** 


### Notes on Preparations for the Sick.

**Diabetic Foods.**—G. VAN ABBOTT AND SONS, London.—It is commonly supposed that foods for diabetics must be absolutely free from starch and sugar. We have examined this excellent series of preparations, which have justly a considerable reputation and are the staff of life to thousands of patients. We append the results, which show that neither of the preparations examined is absolutely free from saccharine matter.

Some little latitude is therefore a necessity, as no diabetic obtains food absolutely free from sugar; hence the modern tendency to allow a limited amount, varying with the condition and capacity of each individual patient.

The following are the results we have obtained from an analysis of some of these foods: On extracting with water and adding solution of iodine in potassium iodide, a deep-blue colour of varying intensity is obtained in each case, which disappears on heating, reappearing on cooling, showing the presence of *starch.* On boiling a portion of biscuit with ten per cent. sulphuric acid to convert starch into sugar, we found the amount of sugar, estimated by the Fehling method, to be—

| | | |
|---|---|---|
| 1. | Caraway Biscuit ... ... | 42 per cent. |
| 2. | Almond Cake ... ... ... | 27 " |
| 3. | Ginger Biscuit ... ... ... | 21 " |
| 4. | Spanish Biscuit... ... ... | 3·375 " |
| 5. | Soya Hispida ... ... ... | 5·625 " |

The Gluten Bread and the Gluten Biscotte both showed traces of starch by the iodine test.

**Cascara Jelly (Horse-shoe Brand). Codeine, Glycerine, and Tangerine Jelly. Edulcorated Castor Oil. Nitrous Elixir.**—C. R. HARKER, STAGG & MORGAN, London.—Samples of these excellent preparations have been forwarded. The **Cascara Jelly** should be an acceptable substitute for the crude bitter extracts of this drug. Some patients cannot swallow capsules, others will not admit that the aromatic preparations are agreeable. A jelly of fruity flavour, with little medicinal taste, and withal decidedly active, must find a somewhat extensive field of utility. The **Codeia Jelly** contains one-eighth of a grain in each drachm. **Castor Oil** is not yet entirely displaced by the newer laxative drugs: the newer preparations of the oil are too good to be overlooked. The **Nitrous Elixir** is introduced as a substitute for Spt. Æth. Nit. B. P. It yields seven times its volume of nitrous oxide gas.

---

**Anti-Asthmatic Cigarettes. Anti-Asthmatic Powder.**— S. KUTNOW & CO., London.—It is always difficult to select from the many powders and cigarettes provided for the asthmatic. Those containing datura and lobelia are often depressing to the heart, although efficient in giving relief to the respiratory difficulty. The Kutnow powder contains neither of these drugs: the cigarettes are well-made, with a mouthpiece; they are pleasant to smoke, they burn with regularity, do not go out, and the ash does not fall in an incandescent state. They have a very pleasant aroma, the smoke is not pungent, and the fumes can be freely inhaled in a concentrated state. They contain no tobacco, are entirely medicinal, and are so innocent that the asthmatic wife might anticipate and prevent the attack by joining her husband in his evening smoking-room social pipe.

---

**"Wincarnis," Crown Imperial Invalid Champagne.**—COLEMAN & CO. LIMITED, Norwich.—The name of Coleman is inseparably associated with medicated wines for the invalid. The well-known triplet of wine, meat, and malt, now known as **"Wincarnis,"** is followed by a twin combination of champagne with the syrup of phosphates formerly known as Parrish's food. It is an agreeable sparkling beverage, with the ordinary characters of a very sweet champagne, but it is not likely to be quoted in the lists of high-class brands or served at dinner parties to candidates for gout.

---

**Coca Wine.**—LORIMER & CO., London.—This is a very agreeable drink which contains a decided flavour of the coca leaf.

---

**Peat Wool.**—THE PEAT INDUSTRIES SYNDICATE LIMITED, London.—This is intended as a cheap and effective substitute for absorbent cotton or wood wool. It is a dark brown fibrous substance, with a pleasant odour, prepared from peat. It readily absorbs fluids when held loosely; but it has the fault of so many other absorbent substances in not proving absorbent when compressed by a bandage. We tried it in a case of pelvic abscess, and found that all discharge remained beneath or trickled out from under it.

---

**Best Olive Oil. Olive Oil Soap. Carbolic Palm Oil Soap. Hospital Antiseptic Soap.**—A. & J. WARREN, Bristol.—This well-known firm of Bristol druggists have sent us specimens of their pure olive oil and of three kinds of soap. The oil is of a beautiful clear colour, and is perfectly free from offensive odour, while the soaps are evidently carefully prepared and pleasant to use. The Hospital Antiseptic Soap contains red iodide of mercury, and is a very cheap and effective preparation for hospital use.

---

**Johnston's Ethereal Antiseptic Soap.**—PARKE, DAVIS & CO., London.—This soap is in the form of a liquid of a pleasant odour. It is easily washed off with water, and is particularly designed for cleansing a greasy surface of skin preparatory to a surgical operation. We have used it, and found it effectual and handy, as it combines the two essentials (ether and soap).

---

**Savar's Cubeb Cigarettes. Savaresse's Sandal Wood Oil Capsules.**—EVANS, LESCHER & WEBB, London.—**Cubeb Cigarettes** are much used in America for hay-fever and catarrh. The specimens sent us are very well made, and constitute a pleasant way of administering an unpleasant drug.

**Savaresse's Capsules** are covered by a double membrane, which generally remains entire until they have passed the stomach, thus avoiding the nauseating effect of the medicine contained in them. Patients who have taken these capsules prefer them to all others.

---

**Antiseptic Jelly. Antiseptic Cream.**—C. J. HEWLETT & SON London. — These preparations are supplied in convenient metallic tubes, and are elegant applications which we can recommend from personal use. The **Jelly** contains 1 in 1,000 of perchloride of mercury, and is a lubricant for surgeons and obstetricians. The **Cream** is a soothing and fragrant application for eczema, intertrigo, or other allied affections. It gives great relief when applied to an inflamed surface.

**Ethyl Choride (Bengué).**—B. Kühn, London.—Dr. Bengué's **Ethyl Chloride** is becoming widely known and highly popular as a local anodyne, as well as a local anæsthetic. It is supplied in glass flasks, fitted with capillary tube or jet and closed with a metallic stopper. When the latter is removed and the flask held in the warm hand, a fine spray is immediately ejected, which will cause complete local anæsthesia in about half a minute. The anæsthesia will last about two minutes, which is long enough for tooth extraction, or many operations in minor surgery. It is decidedly superior to the ordinary method of freezing by ether spray.

---

**First Field Dressings.**—The Liverpool Lint Co., Liverpool. —This is a small packet containing a bandage, a piece of sublimated cotton tissue, waterproof jaconette and gauze. It is intended for first aid on the battle-field, and would form an invaluable item in a soldier's knapsack.

---

**Christia-backed Lint.**—Thomas Christy & Co., London.— This is a lint covered on one surface by an impervious backing, which is not only water-proof, but also spirit-, chloroform-, oil- and grease-proof. We have tried it in the treatment of wounds of fingers, and as a dressing for recently-opened small abscesses, and are not altogether pleased with it. The drawback is that the layer of lint is not thick enough to absorb much fluid, and it therefore needs another piece of lint under it, in which case we cannot see its superiority over a piece of lint covered with oiled-silk.

---

